# Ten tips on how to manage obesity in the presence of CKD

**DOI:** 10.1093/ckj/sfae317

**Published:** 2024-10-21

**Authors:** Nadine Kaesler, Susanne Fleig

**Affiliations:** Department of Nephrology and Hypertension, RWTH University Hospital, Aachen, Germany; Department of Nephrology and Hypertension, RWTH University Hospital, Aachen, Germany

**Keywords:** weight loss diet, chronic kidney disease, personalized nutrition

## Abstract

Patients with chronic kidney disease are frequently facing the challenge of weight reduction. Finding a weight loss strategy is on the one hand essential to reduce the co-morbidity risks in CKD but remains complex due to the metabolic abnormalities with declining renal function. Here, we provide ten tips to support our CKD patients on their journey, focussing on dietary and behavioural habits and health professional supportive therapies.

Having overweight or obesity is a risk factor for chronic kidney disease, high blood pressure, diabetes mellitus [1], metabolism-associated fatty liver disease, cancer, and cardiovascular disease; starting with a body mass index (BMI) >25 kg/m^2^, more weight is associated with increased risk [2]. It pays well to manage weight: many patients need less blood pressure medication and have better kidney function after reaching a normal BMI, on top of lowering their cardiovascular risk long term. Independent of body weight, unhealthy eating patterns are an additional risk factor for CKD [3]: if ultra-processed foods (UPF) account for >30% of the diet, CKD risk is markedly increased [4].

But healthy nutrition is not only CKD prevention; changing one's diet at 40 years of age from an unhealthy to a healthy eating pattern may increase life expectancy up to 10 years, as modelled recently [5].

Helping our patients on their journey to lose weight is potentially a powerful means of primary and secondary CKD prevention; however, the presence of CKD does not ease the task. Healthy diet patterns contain plenty of fruit and vegetables; how to keep potassium levels in place? Many low-carbohydrate diets are high in protein: not an appropriate option in CKD. At the same time, low-protein diets risk malnutrition and sarcopenia, especially when caloric intake is reduced; and sarcopenia and malnutrition go along with bad outcomes. CKD patients need a strategy to lower fat mass while preserving or increasing muscle mass. We have summarized a step-by-step approach for weight loss while keeping in mind the pitfalls of CKD. We know that there are little data on sustainability of long-term behavioural changes as means to produce weight loss; however, healthy lifestyle changes have multiple benefits, the different measures may add up and help to approach the goal.

## DO GET TO KNOW YOUR PATIENT AS A BASIS FOR PERSONALIZED AND PRECISION NUTRITION

Every person is different, and nutrition therapy needs to be personalized to be successful. A good start to establish trust is a welcoming and open environment to get to know our patient.

We need to understand our patient's social and economic situation. Who cooks and prepares the food? Lifestyle changes require a high motivation and family support increases success. If we understand the current diet pattern, we can identify areas with highest leverage; how can we improve nutrition under consideration of individual preferences or allergies?

Food frequencies questionnaires and repetitive 24-h food recalls can evaluate the current diet quality and quantity. Food frequencies questionnaires ask for average consumption of a long list of food items over the past 6 months and need to be adapted to local eating habits. A 24-h food recall asks participants to recall what and how much they have eaten and drunk on the day before, and these recalls should be collected for at least three different non-consecutive days (weekdays and weekend days). Mobile phone apps can register and count diet items during the day, helping to not forget small snacks. For the evaluation of diet quantity and quality, consulting a renal dietician promises competent and valuable input on ‘real life’ vs required energy intake, distribution of macronutrients, and supply of micronutrients in your patient's diet; they can also develop a ‘weight loss strategy’ with the patient with weekly meal plans and changes that lead to lower caloric intake and improved dietary quality to avoid malnutrition.

Also, documenting body weight and waist circumference before start are essential to estimate the personal daily dietary requirements. Repeating these measurements in regular intervals can help to motivate our patients, especially when first results become measurable. These numbers help us to communicate the weight loss goals without using judgemental terms. We further need to consider whether comorbidities also impact the quality of life. Keeping track of improvements of a health-related quality of life indices can keep the patient's motivation up.

## DRINK MOSTLY WATER!

For fluid homeostasis, we need water. Water, unsweetened tea, and coffee contain little to no calories or additives and are therefore healthy to drink. Sweetened beverages as lemonades or coke are UPF [4] that contain sugar or artificial sweeteners as well as phosphate and potassium containing additives and are counterproductive in the context of CKD. Coke flavoured soft drinks contain phosphoric acid, a key player in vascular calcification and cardiovascular disease.

Therefore, soft drinks should be removed from the menu.

Sugar sweetened beverages (SSB) have low nutritional value. They do not contribute to satiety but promote a stronger sensation of hunger [6]. Sugar and other high-glycaemic index carbohydrates are taken up rapidly and trigger insulin release, followed by a drop in blood glucose, hypoglycaemia, and increased appetite. Beverages often contain sucrose and high fructose corn sirup, which have insulin-independent unwanted metabolic effects: fructose intake promotes hyperuricemia, lipogenesis, arginine vasopressin levels and can worsen kidney function [7]. SSB consumption is associated with T2DM, obesity, cardiovascular disease and metabolism-associated fatty liver disease (MAFLD) [8], as well as CKD risk [9], and in CKD with disease progression and all-cause mortality [10].

Beverages with artificial sweeteners are no good alternative: Non-nutritive sweeteners such as aspartame, sucralose, saccharin, or cyclamate can negatively affect the gut microbiome [11] and cause glucose intolerance [12].

Obesity itself can damage the taste system and associates with a depressed sense of taste. To change the dietary habits sustainably, a stepwise reduction of sugar and sweet beverages can help to adapt to the two dimensions of sweet taste: detection thresholds and taste preferences.

## DO ENCOURAGE SLOW AND MINDFUL EATING

Satiation and satiety are complex metabolic processes that involve gastric stretching, endocrine and neuroendocrine signals, and external environmental and behavioural factors. Satiation already starts before eating: during meal preparation, meal quality in the dimensions of flavour, texture, consumer appeal, and nutrient composition is already cognitively processed [13] and a first step towards satiety. Mindfulness can support healthy food choices. Olfactory dysfunction is present in ∼30% of CKD patients [14] and can negatively influence flavour enjoyment but can improve with training [15].

Mindful eating can not only increase the taste experience, but it can also help to keep energy intake lower: satiety signal such as gastric stretching and the secretion of endocrine and neuroendocrine signals take time. By eating slowly, patients can perceive satiety signals and may stop before the plate is empty. Slower portion sizes can help as well, large portion sizes lead to increased energy intake. To not override the satiety signals, also recommend food with lower energy density (such as soups or salads) to start. Eating in front of computer or television should be avoided. Snacking in between meals often provides unnecessary calories and negatively affects insulin signalling.

Remarkably, a decreased body fat mass, in combination with preserved lean body mass, will reduce appetite and further weight loss will become easier [16, 17]. Dietary habits should be changed stepwise without crash diets to prevent long-term weight gain after dieting [17, 18].

## DO ADAPT TO A HEALTHY EATING PATTERN

Changing the proportions of dietary components can have a major impact towards shifting to a healthier diet. This may have only modest influence on body weight, but plenty on cardiovascular outcome: Vegetables, legumes, whole grains, and fruits should make up a large proportion of the ingested daily food [19], and the less processed the food, the better [4]. The 2024 KDIGO guideline [20] recommends a ‘healthy and diverse diet with a higher consumption of plant-based foods compared to animal-based foods and a lower consumption of ultra-processed foods’ [20]. Processed food can be classified into four groups, from minimally processed to ultra-processed [3]. UPF are, for example, soft and energy drinks, instant soups, cakes and cookies, and food additives that are used in the industrial production process [3]. Less processed and more plant-based foods contain more nutrients and less energy than UPF, thereby reducing energy uptake without necessarily changing portion size.

Sodium triggers fluid retention (hypervolemia increasing the weight on the scale) and high blood pressure, which is a problem especially in more advanced CKD stages; WHO recommends <2 g sodium (<5 g salt) per day for the general adult population. Sodium additives are high in processed foods, snacks, sauces, and meat products (>600 mg/100 g [21, 22]). Dialysis centres with a strong focus on sodium restriction and removal report less antihypertensive medication, lower left ventricular hypertrophy, better cardiac function on echocardiography, and lower cardiovascular morbidity [23].

Protein content is a hot topic in nutrition in CKD. Low-protein intake decreases enteral uremic toxin production but comes with a risk of muscle wasting and sarcopenia; CKD by itself is associated with inflammation and protein degradation [24]. For a low-protein diet to be safe, it needs to be accompanied by appropriate energy intake and be monitored by the medical team [25]; in a situation where we aim to lose weight, energy intake will be reduced, and muscle mass needs to be preserved. Mortality in the MDRD very-low-protein cohort was two times higher than control in the long-term follow up [26], and 10-year mortality in patients with mild to moderate CKD >60 years of age is higher in lower protein intake [27]. This risk is increasingly recognized and reflected in current guideline recommendations [20]. To sustain muscle mass, 0.8 g protein/kg of ideal body weight per day are required [28]; using the actual body weight in the setting of obesity can provide excessive amounts of protein. Patients who are trying to lose weight are not metabolically stable (as they aim for a calory deficit), so further protein restriction should be avoided to maintain muscle mass. In advanced kidney disease and on dialysis, higher amounts of protein may be necessary to avoid muscle wasting; also, exercise or physical therapy should be implemented.

## DO IMPLEMENT FIBRE-RICH FOOD

Protein source matters [29]: Protein from plant sources is associated with lower uremic toxin and serum phosphorus levels, and lower mortality [30]. Plants contain dietary fibre, non-digestible carbohydrates that are either metabolized to (beneficial) short chain fatty acids (SCFA) by symbiotic bacteria in the colon, or are water insoluble, indigestible to microbiota and decrease colon transit time while increasing stool volume (laxative effect). SCFA are an energy source for colon epithelial cells and thereby improve the intestinal barrier function, reducing toxin uptake.

Dietary restrictions in advanced CKD rise from fear of hyperkalaemia; however, potassium from fruit and vegetables has a much lower bioavailability than potassium from UPF sources [4, 20]. Additionally, natural potassium sources in food have little influence on hyperkalaemia [31].

CKD patients have a less diverse gut microbiome [32]. Potassium restriction often leads to a low-fibre diet, leaving less substrate for symbiotic bacteria and less SCFA. Intestinal malabsorption allows for more protein in the colon, fostering proteolytic bacteria; also, some blood urea nitrogen is secreted into the colon, feeding urease-producing microbiota; the metabolite ammonium further damages colon epithelium. Lower fibre intake increases gut transit time, allowing for higher uptake of uremic toxins. In sum, higher production (microbiome) and lower excretion of uremic toxins (CKD) speed up CKD progression.

The WHO recommends 25 g of fibre/day [33]. A fibre-rich diet can slow CKD progression [34], and a high fibre intake associates with lower inflammation and all-cause mortality in CKD patients [35]; however, they tend to consume less than half the recommended amount [35].

The major fibre sources are whole grains and legumes, followed by vegetables and fruit [36]. Leftovers can be beneficial due to new fibre components: ‘resistant’ starch results from starch retrogradation after cooking, e.g. in cooked, cold rice [37], potatoes, or pasta, and may reduce blood phosphorous, uric acid, IL6, and indole phenol sulphate in dialysis patients [38]. Potassium and phosphate are less bioavailable from high-fibre sources [19]. A helpful tool to prevent hyperkalaemia and to ensure sufficient supply of fibres, antioxidants and vitamins by maintenance of a low net fixed acid load can be the ‘potassium to fibre ratio’ of food items [39]. Contrarily, a total of 31 approved food additives contain potassium salts that are highly bioavailable [40] and potassium containing additives were present in ∼29% of pre-packaged supermarket products [41].

## INTERMITTENT FASTING TO BOOST THE EFFECTS OF NEW DIETARY PATTERNS

Kidney function, as most physiological functions, follows circadian aspects, such as circadian rhythm of renal excretion, proximal tubules transporters, or renal clock genes [42]. Everyday challenges can disrupt our activity-rest cycle [43] and this may lead to increased energy intake and overall poorer diet quality [44]. Time-restricted eating can affect circadian clock markers, such as the secretion of melatonin and cortisol [45]. Intermittent fasting (IF) was shown to induce significant weight loss and improvement of metabolic diseases, such as diabetic nephropathy [46].

IF comes in different flavours. ‘Periodic’ or ‘alternate day fasting’ means alternating fasting days (no food or sweetened beverage) with days with food [47]; a modified version reduces energy intake on two non-consecutive days per week to 20%–25%, with normal uptake on the other days. Most popular is time-restricted eating: eating only during a fixed 6–8 h time window each day and fasting during the other hours (18:6, 16:8 h) [47], adherence is typically high. The time restriction counteracts a disrupted circadian eating behaviour which associates with obesity [48]. The expression of clock genes can be reset to normal oscillation and glucose control can be improved [49]. IF can improve disorders related to diabetic nephropathy, such as lipid metabolism disorders and hypertension [46]. Observational studies report significantly lower rates of acute kidney injury during the (intermittent) fasting month of Ramadan [50]; for CKD, results are mixed in non-controlled, observational, retrospective studies [47] due to differences in fasting duration, temperature, and fluid restriction. A pilot study in overweight CKD patients showed a high compliance and improvements of the GFR by time-restricted eating [51].

Intermittent fasting and caloric restriction each associate with weight loss, and they can be combined for additive effect. Fasting can improve the cardiovascular outcome [52]. CVD is the major cause of death in CKD [53, 54]. In a rat model of acute kidney injury, fasting led to reductions of oxidative stress, mitochondrial dysfunction, and fibrosis [55]. However, convincing long-term data in humans are still missing, and sustaining this eating pattern may be difficult.

## DON'T FORGET TO MOVE!

For weight loss to happen, we need a setting of negative energy balance. 1 kg of body fat mass equals 7000 calories; to lose 1 kg, we need to burn 7000 calories more than we eat. Muscles are our main organ to spend energy, and physical activity (PA) is necessary to achieve that negative energy balance without losing muscle mass itself. PA can help to lose 1–2 kg extra and has additional benefits: PA by itself has been shown to lower CKD incidence and progression [56–58], and 150 minutes of PA (or more) lower CKD and cardiovascular risk [20]. Motivating our patients to include PA into their schedule is important. Small steps, such as using stairs instead of elevators, having short walking breaks during work, using a step counter or fitness device are a good start. PA may be integrated into daily commutes to work: is there public transportation that can help to reach work with some required walking distances? Is riding a bike an option? Water gymnastics can be very efficient to burn calories and be gentle on the joints at the same time, as surrounding water lowers the weight on the joints. Cardio fitness or physical therapy can help to tailor a training programme to the individual needs and risks. Even in dialysis patients, regular exercise during dialysis was associated with better health outcomes [59].

Especially in the setting of caloric reduction for weight loss, protein supply should be high enough to maintain muscle mass and avoid sarcopenia and malnutrition.

## KETOGENIC DIET (WITH PROFESSIONAL SUPERVISION)

Ketogenic diets are high-fat, minimal-carbohydrate and moderate protein diets that mimic a fasting state by presence of ketone bodies in the circulation due to lipolysis.

Two studies have recently shown that a ketogenic diet is feasible in CKD. In the KETO-ADPKD [60] trial, ADPKD patients, with an average GFR of 84 ± 24 ml/min/1.73 m^2^, on a ketogenic diet (KD) lost 5–6 kg within 12 weeks. The study (a normocaloric ketogenic diet) did not aim for weight loss, and still >70% of the patients in the KD group lost >10% of fat mass in 12 weeks [60]. The investigators used a vegetarian ‘kidney-healthy’ KD [61, 62] with a high proportion of plant-based, low processed food and a low-to moderate protein content of 0.8 g protein/kg BW/d [60], avoiding high-protein load, processed meat products, and choosing more polyunsaturated than saturated fatty acids. Upper daily limits were defined for sodium chloride (7 g/d), phosphorus (700 mg/d), oxalate (100 mg/d), and potassium (4000 mg/d) [60]. The energy in KDs comes 70–90% from fats, the carbohydrate intake is highly restricted. Either a ketogenic ratio of 2:1 to 4:1 of fats to carbohydrates plus proteins is calculated or a fixed intake of carbohydrates is applied, such as 30 g per day [60]. Ketone bodies were measured regularly to assure a ketogenic metabolic state. Given the complexity of this diet, it should be done in collaboration with nutritional therapists and under regular medical supervision.

First clinical studies are examining KDs for weight loss in mild to moderate CKD [63]. We have no data yet on patients with advanced CKD, so caution should be used in higher CKD stages.

KD is discussed controversially in the field [64–66]. Owing to the high use of fat for energy, an increase in LDL is reported and expected, and while it is rationally explainable, it is a known risk factor for atherosclerosis and cardiovascular disease; also, a higher incidence of kidney stones is reported. Long-term data on a kidney-healthy KD and cardiovascular risk are not available, and a KD may be difficult to sustain long term.

Caution is also required in the context of diabetes. Ketone bodies feedback to adipocytes by stimulating insulin secretion, which leads to a suppression of lipolysis and thus ketone body production [51]. Therefore, type I diabetic patients are at especially higher risk of ketoacidosis. Nevertheless, interventional trials showed beneficial effects in type II and type I diabetic patients regarding HbA1c reduction and glucose control [67]. As total cholesterol and LDL may increase under such diets [68], blood lipids should be monitored.

## LET MEDICINE SUPPORT YOU: GLP1-AGONISTS TO THE RESCUE

GLP1 receptor agonists (GLP1RA) such as semaglutide, liraglutide, and the combined GIP/GLP1RA tirzepatide have revolutionized pharmacotherapy for obesity; initially developed for diabetes treatment, they showed better glycaemic control as well as lower body weight in treated patients and have been approved for obesity management. Via activation of proopiomelanocortin and inhibition of neuropeptide Y in the hypothalamic arcuate nucleus, they decrease hunger and increase satiety, thereby reducing *ad libitum* energy uptake by ∼30%. Patients typically reach a weight loss of ∼15% body weight within a year. They require careful nutritional management, as necessary macro- and micronutrients need to be included in the lower nutritional intake.

They are not only good for diabetes and obesity management: GLP1-RA reduce major adverse cardiovascular events by 14%, all-cause mortality by 12%, hospital admission for heart failure by 11%, and composite kidney outcome by 21% [69]. The FLOW trial [70] (NCT03819153) was designed as a randomized, double blind, multinational phase 3b kidney outcomes trial for semaglutide in patients with diabetes mellitus and CKD (eGFR > 25 ml/min, <75 ml/min), and enrolled >3500 patients with DM2 and CKD. It was stopped early due to efficacy; in March 2024, the company announced that once weekly semaglutide 1 mg reached its primary endpoint by reducing kidney disease progression as well as cardiovascular and kidney death by 24% compared to placebo and reached superiority in its secondary outcome parameters [60]. GLP1RA are expected to become a cornerstone in CKD management in the next years, especially in overweight patients. However, access to these drugs today is difficult for patients without diabetes and in higher CKD stages.

SGLT2 inhibitors (e.g. dapagliflozin or empagliflozin) prevent tubular reabsorption of glucose from urine and are approved for diabetes, heart failure and CKD treatment (to start while eGFR is >20 ml/min/1.73 m^2^); due to the induction of glucosuria, patients may lose on average 2–4 kg.

Bupropione/naltrexone is a fixed drug combination of an aminoketone antidepressant and an opioid antagonist approved for weight loss, but not recommended when kidney function is reduced due to higher serum concentrations and more frequent adverse effects.

## IF YOU CANNOT REACH A HEALTHY BODY WEIGHT WITH ALL OTHER MEASURES: BARIATRIC SURGERY CAN IMPROVE CVD OUTCOME

When CKD progresses, patients with obesity have reduced access to kidney transplantation, and even dialysis access may be an issue [71]. If all prior measures fail or do not show sufficient effect, metabolic surgery is an effective option for weight management, reaching a weight loss of about 20%–25%. A case-control-study in patients with CKD and obesity suggests a 58% lower risk of a GFR decline >30% after bariatric surgery [72]. Given that surgery always comes with risk, and that patients with obesity and CKD are at high risk of perioperative complications, surgery is an option if all other strategies have not shown sufficient effect. Metabolic surgery effectively lowers mortality in patients with obesity [73, 74].

The principal methods are vertical sleeve gastrectomy and Roux-en-Y gastric bypass surgery; vertical sleeve gastrectomy having a lower perioperative morbidity, and Roux-en-Y gastric bypass reaching higher weight loss and less gastroesophageal reflux disease.

## CONCLUSION

Currently, several challenges remain. For one, GLP1-agonist demand exceeds supply by far, and people do not receive therapy even when prescribed; companies are working on increasing manufacturing capacities. If, for which indication and under which conditions therapy costs will be covered by insurances/the healthcare system is still under debate in different countries.

For dietary interventions such as KD, we lack data on higher CKD stages and long-term outcome data given the concern about cardiovascular endpoints. For all described interventions, we know that there are little data on sustainability of long-term behavioural changes as means to produce weight loss.

**Figure 1:**
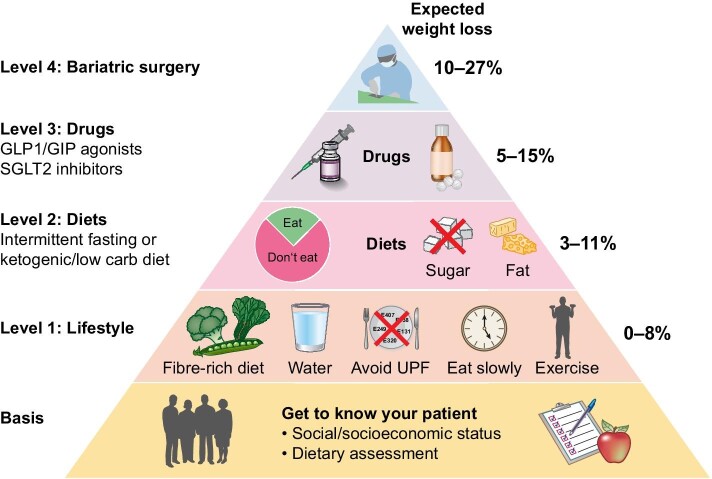
How to lose weight in the context of CKD.

Weight management in patients with obesity and CKD is a complex and interdisciplinary challenge. We have summarized our step-by-step approach in Fig. 1. Nephrologists should enlarge the therapeutic team and include nutritional experts, physical therapists, psychologists, diabetologists, and surgeons to reach a significant weight loss. For all day management and support, the involvement of the whole family is crucial. Changing habits towards a healthy diet has long-lasting benefits beyond weight loss. However, long-term data on weight management is scarce. Not every item in this list will lead to significant weight loss by itself, but they can have additive effects when combined, which may be individually different in scale. Sustainable lifestyle changes helping with weight loss and CKD prevention are always a team effort.

## Data Availability

There are no new data associated with this article.
